# Supplementary material for: The Narrowed Internal Auditory Canal: A Distinct Etiology of Pediatric Vestibular Paroxysmia
Source: J Clin Med. 2022 Jul 25;11(15):4300. doi: 10.3390/jcm11154300 (PMC9332349; doi:10.3390/jcm11154300)
Supplement: Supplementary file 1 [file jcm-11-04300-s001.zip › jcm-1806037-supplementary.pdf]

Supplementary Data

HRCT of temporal bones (IAC sections), for the right and left ear, in both axial and coronal plans of Group I patients (1 to 8). A: Right ear, axial plane, B: Left ear, axial plane, C: Right ear, coronal plane, and D: Left ear, coronal plane.

Note that these figures show uni- or bilateral narrowing of the IAC. Narrowing can be visualized in both axial and/or coronal plans, more pronounced in the coronal plans suggesting a developmental process.

1:

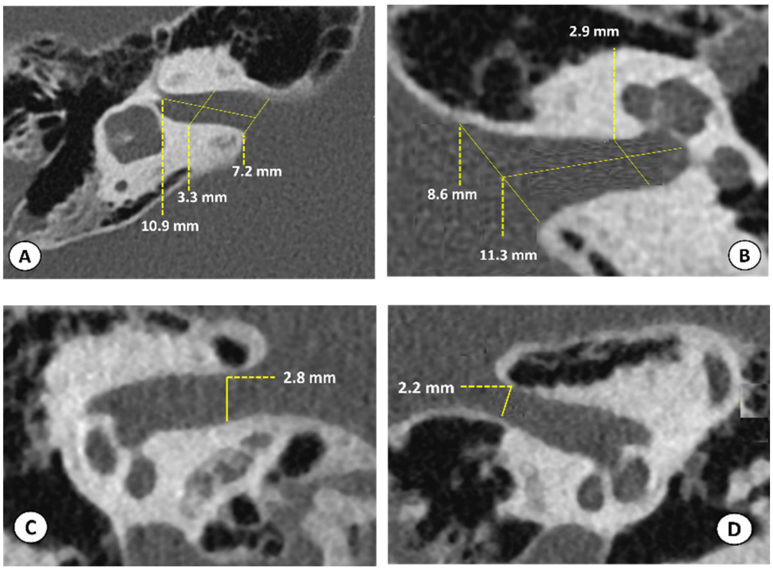

2:

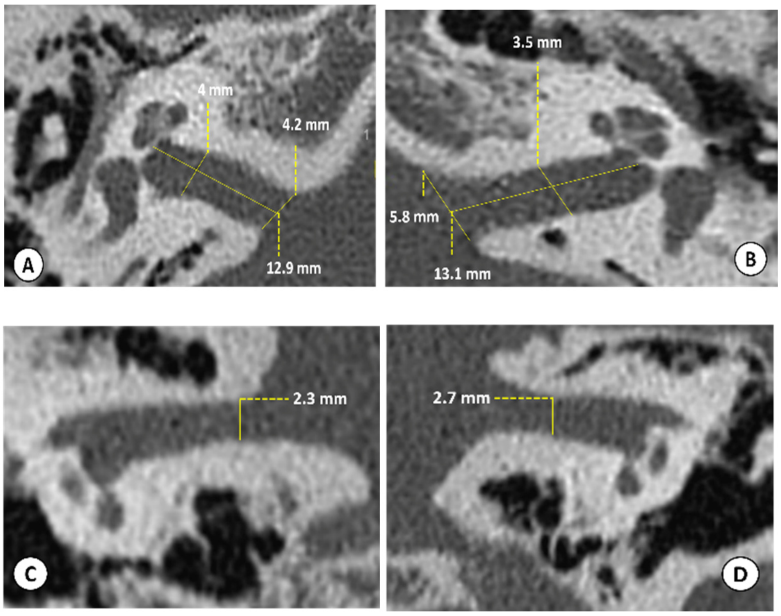

3:

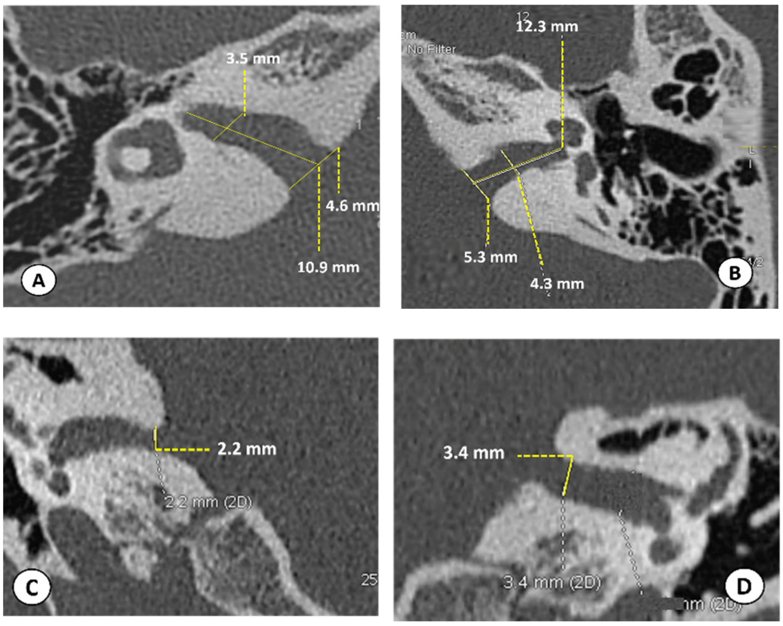
3.5 mm
4.6 mm
10.9 mm
A
12.3 mm
No Filter
5.3 mm
4.3 mm
B
2.2 mm
2.2 mm (2D)
25
C
3.4 mm
3.4 mm (2D)
D

4:

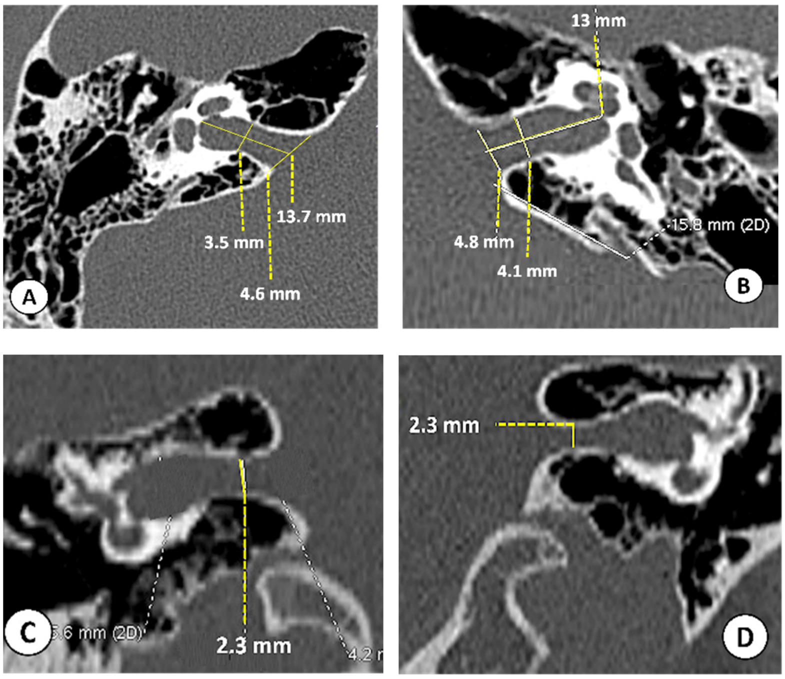
13.7 mm
3.5 mm
4.6 mm
A
13 mm
15.8 mm (2D)
4.8 mm
4.1 mm
B
2.3 mm
C
2.3 mm
D

5:

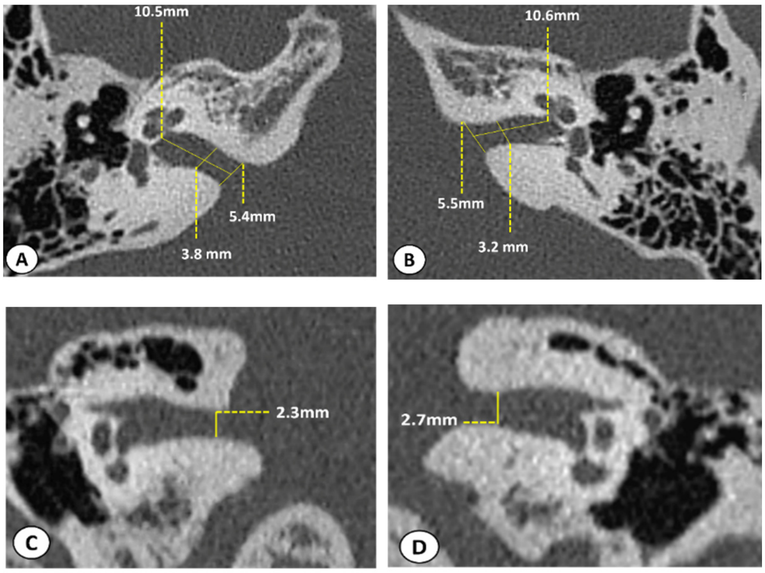
10.5mm
5.4mm
3.8 mm
A
10.6mm
5.5mm
3.2 mm
B
2.3mm
C
2.7mm
D

6:

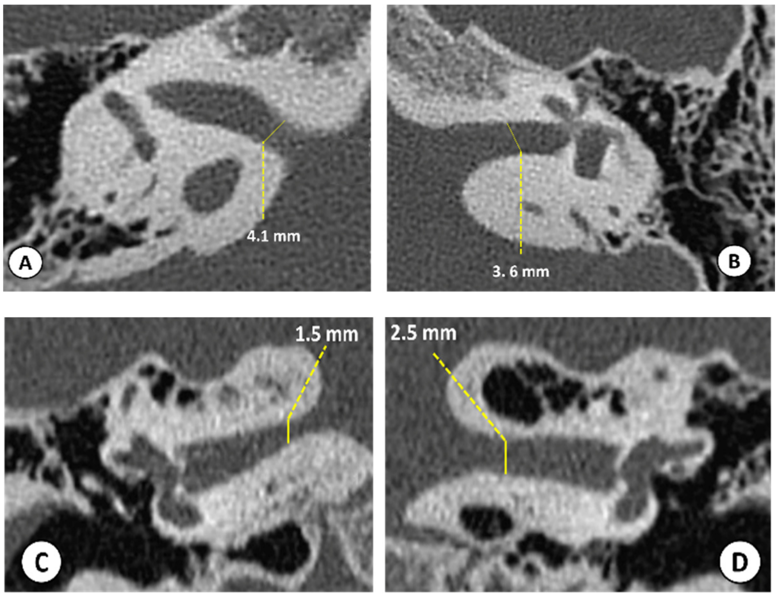
4.1 mm
A
3. 6 mm
B
1.5 mm
C
2.5 mm
D

7:

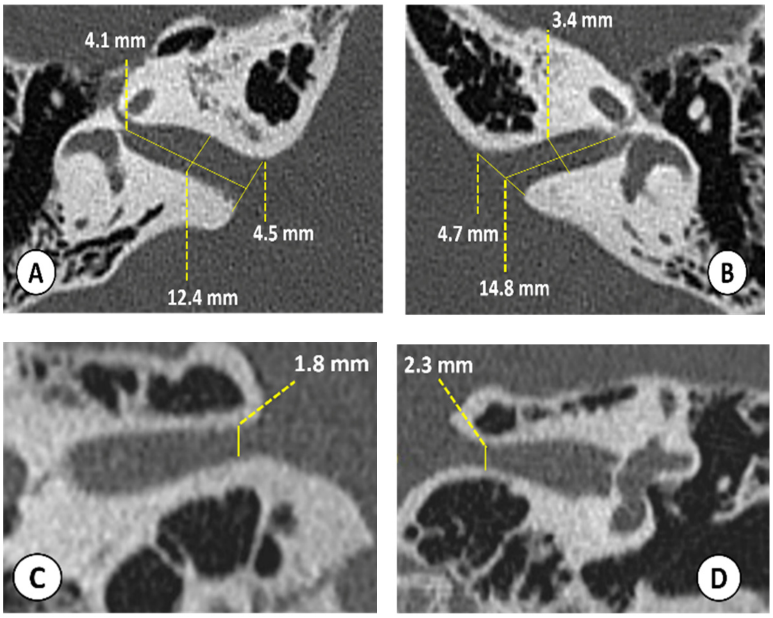
4.1 mm
4.5 mm
12.4 mm
A
3.4 mm
4.7 mm
14.8 mm
B
1.8 mm
C
2.3 mm
D

8:

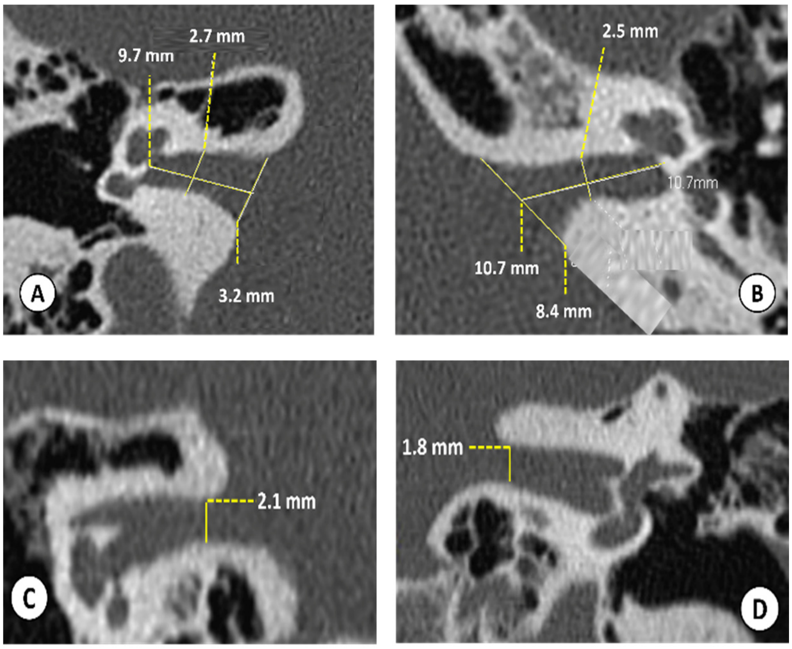
2.7 mm
9.7 mm
3.2 mm
A
2.5 mm
10.7mm
10.7 mm
8.4 mm
B
2.1 mm
C
1.8 mm
D
